# Differences in beta-lactam and penicillin allergy: Beyond the West and focusing on Asia-Pacific

**DOI:** 10.3389/falgy.2022.1059321

**Published:** 2022-11-22

**Authors:** Hugo W.F. Mak, Maegan H.Y. Yeung, Jane C.Y. Wong, Valerie Chiang, Philip H. Li

**Affiliations:** ^1^ Division of Rheumatology and Clinical Immunology, Department of Medicine, Queen Mary Hospital, The University of Hong Kong, Hong Kong, China; ^2^ Division of Clinical Immunology, Department of Pathology, Queen Mary Hospital, Hong Kong, China

**Keywords:** penicillin, allergy, Asia-Pacific, West, beta-lactam

## Abstract

Beta-lactam (BL) antibiotic “allergy” labels are common, but often overdiagnosed. Although much research has been focused on the BL allergy and the delabelling process in the West, studies from other parts of the world remain sparse. This review outlines the contrasting global epidemiology, shifting clinical practices and disparities of BL allergy in the Asia-Pacific region compared with the West. Innovative strategies to overcome barriers in BL allergy workup are discussed and potential directions for future research and service development are also proposed.

## Introduction

Antibiotics can result in a plethora of adverse drug reactions presenting as both immune-mediated (i.e., allergies) and non-immune mediated manifestations (i.e., intolerance) (1). Although most adverse reactions are non-immune mediated, they are frequently mislabelled as “allergies” and these labels often remain lifelong without further evaluation. Owing to their widespread use as first-line therapy for most infections, beta-lactam antibiotics (BL) remain as one of the leading culprits of drug “allergy” (2–4). However, most BL allergy labels are incorrect with only 5%–15% confirmed to be genuine after allergological evaluation (5, 6). Even among genuine BL allergic patients, sensitivities diminish over time and only around 10% of individuals remain sensitized after 10 years following avoidance (7, 8).

Among those labelled with BL allergy, the obligatory use of less effective and more harmful second-line antibiotics is associated with poorer clinical outcomes such as higher admission rates, in-hospital mortality, and risk of infection from multidrug resistant organisms (including *Clostridioides difficile*, methicillin-resistant *Staphylococcus aureus*, and vancomycin-resistant *Enterococcus*) (9–11). These adverse outcomes are especially accentuated among vulnerable and immunocompromised individuals, such as patients with underlying immunological diseases and the elderly (9, 11, 12). In the era of COVID-19, patients with BL allergy labels were shown to have higher rates of ICU admission, acute respiratory failure, need for mechanical ventilation and overall mortality (13, 14).

From a public health perspective, suboptimal therapy because of mislabelled BL allergy can be a waste of efficiency and healthcare resources. BL allergy labels are also associated with higher mean antibiotic costs, as well as higher cost during hospital admission and discharge, wherein lengthier hospital stays increase healthcare expenditure up to ten-fold (15). BL allergy delabelling has been shown to effectively reduce prescriptions of second-line antibiotics and promote re-uptake of first-line penicillins in delabelled patients (16–18). From Australian experience, antibiotic stewardship was enhanced with increased narrow-spectrum penicillin usage (19), with a low risk of anaphylaxis recurrence (20). Economically, delabelling has been shown to generate potential savings of $2000USD per patient-year and switching from other broad-spectrum antibiotics to BL has also been found to lower inpatient and outpatient prescription costs by up to $609USD and $193USD per patient respectively (15, 21). Studies on the Australian Penicillin Allergy Delabelling Program have also demonstrated cost-savings of $20.51 per effectively delabelled patients, even significantly reducing costs as compared to outpatient testing strategies (22).

Identifying genuine BL allergy, i.e., delabelling the mislabelled, is thus vital for healthcare optimization at both individual and institutional levels. However, existing practices on BL allergy and delabelling are widely substantiated based on inputs and experience from “the West”, which includes Europe, North America, Australia and New Zealand in this article, warranting attention to this predicament from an Asia-Pacific perspective.

## Epidemiology: East vs. West

The prevalence of BL allergy varies across regions. Global estimates derived primarily from United States and European studies report an approximate 10% (8%–15%) prevalence within the general population (23, 24). The reported rate of BL allergy labels in hospitalized patients is even higher, ranging from 13%–25% (3, 4, 25–28). Within the BL group, penicillins accounts for most allergic reactions and the prevalence of penicillin allergy labels is often quoted as 8%–10% (3). For cephalosporins, an United States study reported a baseline prevalence of suspected cephalosporin allergy history of 0.9%, and a rate of new reports of cephalosporin allergy as 0.5% per treatment course (29). In comparison, allergies to carbapenem or monobactam are much rarer, with respective prevalences of only 0.007% and 0.001% (30).

Across Asia-Pacific countries (excluding Oceania unless otherwise specified, same hereafter), the regional prevalence of BL allergy labels in both the general population and hospitalized patients are generally lower although the disease burden is still considerable. Regional figures in hospitalized patients from mainland China, Hong Kong and Japan range from 4% to 5.6% (31–33). In Hong Kong, although the point prevalence of BL allergies was only 2% among the general population, population-wide data demonstrated a cumulative incidence of over 100 per 100,000 population (5).

Furthermore, there is a higher rate of documented allergic reactions to second-line broad-spectrum BL in Asia-Pacific. A study comparing patients referred for suspected BL allergies in Hong Kong and the United Kingdom found significantly more referrals in the Hong Kong cohort for suspected hypersensitivity to broad-spectrum antibiotics, including amoxicillin-clavulanate, piperacillin-tazobactam and meropenem (33). Recent studies also show a marked increase in reported allergies to piperacillin-tazobactam in Hong Kong, likely attributable to a more-than 150% increase in local prescription rates between 2015 and 2019 (34). Nonetheless, the pervasiveness of broad-spectrum antibiotic prescription and allergy labels are not unique to Hong Kong but also other Asia-Pacific territories. Piperacillin-tazobactam and cefoperazone-sulbactam rank among the commonest causative agents for BL hypersensitivity in India (35); and broad-spectrum antibiotics such as third generation cephalosporins and piperacillin are top culprits for BL anaphylaxis in Korea (36).

Reasons for these observed regional variations are likely multifactorial, whether it be more robust electronic health record documentation (37), or genuine biological ethnic-specific differences. Historically, certain high-risk HLA alleles were identified to be associated with carbamazepine and allopurinol-induced drug allergy among Asian patients, but not BL or anti-microbials (38, 39). Interestingly, a recent Thai study found HLA-B*48:01 to be associated with immediate-type reactions to BL, whereas HLA-C*04:06, HLA-C*08:01 and HLA-DRB1*04:06 were associated with delayed reactions (40). In contrast, studies from the West have reported HLA-B62 as a possible risk factor for drug reaction with eosinophilia and systemic symptoms to piperacillin/tazobactam (41). From a perspective of antimicrobial stewardship, these may also be related to the regional differences in antibiotic prescription patterns, such as higher rates of over-the-counter availability of antibiotics (42), local microbial sensitivity and resistance patterns prompting need for broad-spectrum antibiotics and adherence to regional antimicrobial stewardship programs (43, 44).

## Differences in skin testing and sensitization

Sensitization patterns to penicillin allergic determinants also vary through time and space. For example in Australia, the sensitization rate to only penicillin determinants (benzylpenicilloyl polylysine (PPL) ± minor determinant mixture (MDM) ± benzylpenicillin (BP)) was 9.5% while in the UK, only 8% of patients were monosensitized to PPL or MDM (45). In a 13-year United States study, the rate of positive penicillin skin tests dropped from more than 10% to below 5% (46). Another recent Spanish study also showed a progressive decrease in sensitization rate to penicillin determinants from 57.6% to 22.1% over the last 25 years, echoing past European findings that the diagnostic sensitivity of PPL, MDM and BP has dropped to only 20% and omission of these determinants in skin test was justified (47–49). Differences in sensitization patterns between Asia-Pacific and European countries have been directly compared. A cross-sectional population study in Hong Kong found that 20.4% and 10.2% of patients were only monosensitized to PPL and MDM respectively (5). Therefore, although omission for penicillin determinants have been popularized in selected Western populations, we advocate that PPL and MDM should remain components of the routine panel testing in selected populations – especially in light of high rates of PPL/MDM monosensitization at least among predominant Chinese populations (5).

Variability in the use and availability of skin test reagents will also determine differences in sensitization patterns. For example, in the United States, there is no commercially available MDM so many centers did not comprise that as a component in penicillin skin test but only use PPL and BP (50). Even for PPL, its commercial form used to be withdrawn from the United States market since 2004, until it regained full approval from the United States Food and Drug Administration in 2009, significantly impacting clinical practice as well as resultant statistics and studies during the period (46, 51). Alternatively, in Europe and Hong Kong, PPL, MDM, BP, amoxicillin are commercially available and routinely included in skin testing for penicillin allergy (5, 52, 53). This is, however, not the common case in other Asia-Pacific regions. In a study surveying 13 countries in the Asia-Pacific (with Australia included in the study) regarding their diagnostic practices in drug allergy, although 100% of them performed skin test, only 60% of them had access to commercial penicillin kit of PPL and MDM (54).

Furthermore, testing patterns may reflect regional policy differences. For instance, regulations of mainland China mandate routine intradermal test prior to penicillin prescription even in patients with no clinical history of penicillin-induced hypersensitivity reaction (32, 55). Concernedly, screening by skin tests irrespective of clinical history can produce false positive results, creating unnecessary healthcare burden despite good intentions.

## Identifying roadblocks and innovating the practice

Severely limited allergy services and overburdened medical infrastructure seems ubiquitous amongst many Asia-Pacific countries. While the Asia-Pacific represents the majority of the world population and likely the biggest burden of mislabelled “allergy”, there remains a limited supply of Allergists to meet the overwhelming demand (56). Severely low Allergist-to-population ratios are observed even in high-income locales, such as Hong Kong, with each Allergist serving up to 1.17 million population (56). Additional roadblocks in optimizing the efficacy of antibiotic allergy delabelling in resource-limited settings include impeded access to laboratory facilities and reagents, as well as paucities in territory-specific drug allergy guidelines or recommendations (39, 54). It is not possible nor efficient to rely on Allergists alone to tackle the huge burden of BL allergies, therefore innovative and novel strategies have been introduced to facilitate penicillin allergy workup and delabelling in recent years especially in the Asia-Pacific region.

### Risk stratification

A popular and important tactic adopted in many clinical settings is to stratify and triage patients into different groups according to their risk of a genuine allergy. Various clinical predictors have been validated to identify low, medium and high-risk features of genuine BL allergy (18, 33, 45, 53, 57). Studies have shown that risk triage by a comprehensive history alone purports an excellent negative predictive value for low-risk cases which is comparable to skin testing (45, 58, 59). Increasingly so, there is a trend towards using direct drug challenge (usually with oral amoxicillin) in low-risk cases without the need for skin tests (60, 61). Additionally, prioritizing special populations that have imminent need for BL have also been advocated. For example, prioritizing patients with suspected BL allergy pre-operatively in elective orthopedic and obstetric operations have been shown to reduce economic burden, alter antibiotic choices and reduce hospitalization (62–66). At the moment, the risk stratification programs in many Asian locales are still in their infancy and bear striking similarities to Western protocols since both of which are largely based on the research findings in the West (67).

### Multidisciplinary collaborations

With proper risk stratification, further drug allergy workup strategies may be adopted to incorporate a multidisciplinary team with collaboration between Allergists, non-allergists and allied health professionals. Various clinical models that have gained popularity in the past decade include multidisciplinary collaborations with pharmacists, nurses, and non-allergist physicians to implement BL allergy workup among low-risk patients (16–18, 68). With appropriate guidance and training, non-allergists have shown to independently evaluate low-risk cases and conduct delabelling. For example, the Hong Kong Drug Allergy Delabelling Initiative (HK-DADI) has published consensus statements to guide penicillin allergy testing by non-allergists and delabelling is now performed by non-allergists in various “Spoke” Clinics across Hong Kong supported by an Allergist in the “Hub” under a “Hub-and-Spoke” model (53). In fact, experience from HK-DADI has demonstrated that a nurse-led, protocol-driven evaluation was not only safe and effective in penicillin allergy delabelling, but led to an even higher rate of future penicillin use following delabelling and mitigated the need for unnecessary skin testing (18).

### Telemedicine

There is great potential for telemedicine growth in the implementation of delabelling, especially in Asia-Pacific regions where accessibility to Allergist services and facilities may be limited (69). Telemedicine has been used to facilitate Allergist verification of skin tests performed by trained assistants, as well as review and identify patients appropriate for in-consult allergy evaluations (70). These telemedicine-based delabelling program for adult and paediatric patients demonstrated successful antibiotic de-escalation, savings in cost, and reduced active physician time while offsite (70, 71). These programs also report high satisfaction rates, with the majority of patients rating the experience as comparable to in-person encounters (69, 72). However, still suboptimal internet and mobile phone penetration rates as compared to developed countries, drastic urban-rural disparities and integration into existing healthcare systems remain challenges for telemedicine implementation in certain Asia-Pacific nations (73, 74).

## Future steps in connecting the Asia-Pacific and West

The epidemic of BL allergy overdiagnosis is a global problem. More robust epidemiological data to determine and understand the burden and differences of BL allergy, especially in Asia-Pacific, are urgently needed. The outcome and enduring impact of delabelling should not just be limited to the delabelling process and it is important to recognize the clinical, psychosocial, and economic impacts beyond the initial delabelling process. Important data long-term clinical outcomes, which are especially scarce among Asian populations, include patterns of microbial resistance, patient quality of life and overall cost-effectiveness in the years following delabelling. Multi-centre, multi-cultural, international prospective studies are needed for better representation in the Asia-Pacific region.

As mentioned, HLA genetic variations might partially explain the discrepancies between BL allergies between Asia-Pacific and the West and may carry powerful diagnostic potential. However, regional differences in HLA-gene frequency, accessibility of screening facilities, and local availabilities of drug alternatives for high-risk individuals would likely affect the cost-effectiveness and feasibility of prescreening for BL allergy by HLA-genotype in different countries (67). Large inter-ethnic studies would be required to confirm these associations and investigate the potential role of HLA-based strategies for BL allergy workup in the future.

Nonetheless, various large-scale studies within the Asia-Pacific region have demonstrated clinical and economic benefits of BL allergy delabelling. The gains from successful BL allergy delabelling programs demonstrated in these Australian studies provide forays into the potential benefits that could be generated across the Asia-Pacific with widespread implementation of BL delabelling programs in the region.

In conclusion, overdiagnosed BL allergy is a significant public health challenge to be tackled globally. In view of the substantial geographical differences, it is in urgent need for Asia-Pacific to establish more evidence and customize its own delabelling practice to best fulfil its huge and unique demand. Collaborations among disciplines and countries are direly called for. After all, it is the effort and responsibility of every one of us to tackle the global burden of misdiagnosed BL allergies.
